# Gender differences in the association between insulin resistance and chronic kidney disease in a Chinese population with metabolic syndrome

**DOI:** 10.1186/s13098-022-00956-0

**Published:** 2022-12-02

**Authors:** Chieh-An Lin, Wen-Cheng Li, Szu-Yu Lin, Yi-Chuan Chen, Wei Yu, Hsiung-Ying Huang, Xue-Jie Xiong, Jau-Yuan Chen

**Affiliations:** 1 Department of Family Medicine, Chang Gung Memorial Hospital, Linkou Branch, Taoyuan, Taiwan; 2 College of Medicine, Chang Gung University, Taoyuan, Taiwan; 3 Department of Health Management, Xiamen Chang Gung Hospital, Xiamen, China; 4 Department of Pulmonary and Critical Care Medicine, Xiamen Chang Gung Hospital, Xiamen, China; 5 Department of Oncology, Xiamen Chang Gung Hospital, Xiamen, China

**Keywords:** Chronic kidney disease, Insulin resistance, HOMA-IR, Metabolic syndrome

## Abstract

**Background:**

Insulin resistance (IR) was reported to be associated with renal function impairment, but little is known about the gender difference. Hence, our study aimed to investigate the relationship between IR (estimated by the homeostasis model assessment of IR (HOMA-IR) index) and chronic kidney disease (CKD) in a Chinese population with metabolic syndrome (MetS) and discern whether there was any gender disparity or not.

**Methods:**

This retrospective cross-sectional study enrolled 13,638 men and 10,450 women who received health examinations from 2013 to 2016 at Xiamen Chang Gung Hospital. Among the participants, 3,253 men (64.3%) and 1,808 women (35.7%) who had MetS and met the inclusion criteria were included for analysis. Spearman’s correlation was conducted to analyze the relationship between HOMA-IR and cardio-metabolic risk factors. Multivariable linear regression was analyzed to explore the relationship between HOMA-IR and cardio-metabolic variables. Logistic regression analysis was performed to assess the association between HOMA-IR and CKD.

**Results:**

The median HOMA-IR and prevalence of CKD was 2.2 and 11.31%, respectively, for men and 2.09 and 15.93%, respectively, for women. In multivariable linear regression analysis, HOMA-IR was significant associated with estimated GFR, albumin/creatinine ratio in men. Multivariable logistic regression revealed a significant difference between HOMA-IR value and the prevalence of CKD in men but not in women (odds ratio in male = 1.21; 95% CI 1.14–1.28, p ≤ 0.001; odds ratio in female = 1.01; 95% CI 0.99–1.02, p = 0.38).

**Conclusions:**

HOMA-IR was independently associated with CKD among men with MetS but not in women.

## Background

Chronic kidney disease (CKD) is a worldwide public problem which cause huge economic burden to healthcare systems. CKD is associated to cardiovascular disease (CVD) and an increased risk for all-cause and cardiovascular mortality [1]. The mean (95%CI) global prevalence of CKD was 13.4% (11.7–15.1%) and it increased with age [2]. Sexual dimorphism in kidney disease was noted in previous studies. Arguably, CKD was more prevalent in women than in men [2] but the progression of renal disease is faster in men than in women [3]. However, the role of sex plays in the renal physiology and pathology remains unclear.

Metabolic syndrome (MetS) is a cluster of endogenous risk factors, including atherogenic dyslipidemia, elevated blood pressure (BP), elevated plasma glucose, abdominal obesity, insulin resistance(IR), and is commonly seen in patients with CKD [4, 5]. Patients with MetS are at an increased risk of CKD, atherosclerotic cardiovascular disease and type 2 diabetes mellitus [4, 5].

IR has a strong relationship with MetS [6] and is associated with increased risk for CKD [7, 8]. IR is an early alteration associated with CKD; it is apparent when the glomerular filtration rate (GFR) is still within the normal range [9]. In nondiabetic individuals aged  ≥ 20 years, the prevalence and incidence CKD progressively increased with an increase in IR [7, 8]. IR also accelerates progression toward kidney failure [10, 11] by several mechanisms, such as sodium retention, sympathetic nervous system activation, and downregulation of the natriuretic peptide system [9]. Some glucose metabolism biomarkers, such as homeostasis model assessment of IR (HOMA-IR) index, Glycated albumin and Glycated hemoglobin are found associated not only IR but also CKD [7, 12–16].

Therefore, this study aimed to explore the association between IR (estimated by the HOMA-IR index) and CKD, especially about the gender differences, in a relative high risk Chinese population who had MetS.

## Materials and methods

### Study design and population

In this cross-sectional study, 13,638 men and 10,450 women (aged  ≥ 18 years old) of Chinese ethnicity who received health examinations from 2013 to 2016 at Xiamen Chang Gung Hospital in Xiamen city in China were considered for enrollment. The exclusion criteria were as follows: current pregnancy, incomplete data, CKD stages 4–5, end-stage renal disease, presence of a chronic disease that may affect the metabolic status (e.g., thyroid or hypothalamic disease, chronic hepatitis, cirrhosis, malignant tumor, kidney transplants, severe urinary tract obstruction, renal cancer, and glomerulonephritis), or receiving nephrotoxic agents (e.g., nonsteroidal anti-inflammatory drugs (NSAIDs), aminoglycoside antibiotics, chemotherapeutic agents, nephrotoxic herbal medicine). Participant data were collected from their medical examination records. The study was validated by the Institutional Review Board of Xiamen Chang Gung Hospital.

### Data collection and measurement

During the health checkups, well-trained nurses administered a standardized questionnaire to the participants. The questionnaire consisted of information on physiological conditions, medical history, and current medications. Detailed anthropometric measurements was performed, including waist circumference (cm) (WC), body weight (kg), body height (cm) and BP (mmHg). Body height and weight were measured using calibrated meters and scales. Body height was measured without shoes and with the participants standing erect with feet together, looking forward. Body weight was measured with participants wearing light clothing. Body weight was recorded to the 0.1 kg, and body height was measured to the 0.5 cm. Body mass index (BMI) (kg/m^2^) was calculated as follows: body weight/(height)^2^. WC was measured between the midpoint of the lowest rib and the iliac crest with feet 25–30 cm apart. BP was measured using a standardized automated sphygmomanometer placed on the participant’s right arm after at least 10 min of rest. Three consecutive measures of BP with an interval of at least 5 min were taken on the same arm. The lowest of the three measurements was collected for the analysis. Mean arterial pressure (MAP) was estimated as (2/3) × diastolic BP + (1/3) × systolic BP.

After at least 12 h of overnight fasting, patients’ venous blood samples were collected for fasting blood glucose (FBG, mmol/L), total cholesterol (TC, mmol/L), high-density lipoprotein cholesterol (HDL-C, mmol/L), low-density lipoprotein cholesterol (LDL-C, mmol/L), triglyceride (TG, mmol/L), serum creatinine (Cr, μmol/L), and insulin (mIU/L), which were determined by enzymatic, spectrophotometric, or colorimetric methods. Urine albumin and creatinine were also measured.

The estimated GFR (eGFR) based on creatinine level was calculated using the Chronic Kidney Disease Epidemiology Collaboration (CKD-EPI) Equation [17]:

$${\text{GFR }} = { 141 } \times {\text{ min}}\left( {{\text{Scr}}/\kappa ,{1}} \right)^{\alpha } \times {\text{ max}}\left( {{\text{Scr}}/\kappa ,{ 1}} \right)^{{ - {1}.{2}0{9}}} \times \, 0.{993}^{{{\text{Age}}}} \times { 1}.0{18 }\left[ {\text{if female}} \right] \, \times { 1}.{159 }\left[ {\text{if black}} \right]$$

Scr is serum creatinine (mg/dL), κ is 0.7 for females and 0.9 for males, α is −0.329 for females and −0.411 for males, min indicates the minimum of Scr/κ or 1, and max indicates the maximum of Scr/κ or 1.

CKD was staged according to the Kidney Disease Outcomes Quality Initiative (K/DOQI) [18] on the basis of the eGFR and proteinuria measures: stage 1 (eGFR ≥ 90 mL/min/1.73 m^2^ with albuminuria), stage 2 (eGFR 60–89 mL/min/1.73 m^2^ with albuminuria), stage 3 (eGFR 30–59 mL/min/1.73 m^2^), stage 4 (eGFR 15–29 mL/min/1.73 m^2^), and stage 5 (eGFR < 15 mL/min/1.73 m^2^). On the basis of the recommended albumin/creatinine ratio (ACR) values, albuminuria was classified as normoalbuminuria (ACR < 30 mg/g Cr), microalbuminuria (ACR 30–299 mg/g Cr), or macroalbuminuria (ACR ≥ 300 mg/g Cr) [19].

According to the National Cholesterol Education Program Adult Treatment Panel III (NCEP-ATP III) criteria with modification on WC cutoff for Asian population [4], MetS was defined as the presence of at least three of the following five criteria:

(1) Abdominal obesity (WC ≥ 90 cm for men and  ≥ 80 cm for women), (2) high BP (a systolic BP ≥ 130 mmHg or diastolic BP ≥ 85 mmHg, taking antihypertensive drugs, or already diagnosed with hypertension), (3) hyperglycemia (FBG ≥ 5.6 mmol/L, taking antihyperglycemic agents, or diagnosed with diabetes mellitus), (4) decreased HDL-C (< 1.03 mmol/L for men and  < 1.29 mmol/L for women or taking hypolipidemic agents), and (5) high serum TG (≥ 1.7 mmol/L or taking drugs used to treat hypertriglyceridemia).

IR was evaluated using the HOMA-IR index, which was calculated as follows: fasting insulin (mIU/L) × FBG (mmol/L)/22.5 [20].

### Statistical analysis

The continuous data are shown as medians [Q1, Q3] and compared using Mann–Whitney U test. The categorical data, including albuminuria (ACR ≥ 30 mg/g Cr) and CKD, are shown as n (%) and compared using the Chi-square test. Spearman’s correlation was performed to analyze the correlation between HOMA-IR and cardio-metabolic risk factors. Multivariable linear regression was conducted to explore the relationship between HOMA-IR and cardio-metabolic risk factors (age, the waist-to-height ratio (WHtR), MAP, TG, HDL-C, eGFR, ACR). To investigate the association between cardio-metabolic risk factors which were related to metabolic syndrome (HOMA-IR, age, WC, WHtR, MAP, TG, HDL-C, FBG) and CKD prevalence, logistic regression analyses was conducted with and without covariates adjustment. We did not include WC and FBG into the multivariable logistic regression models due to the high variance inflation factor of WC and WHtR, FBG and HOMA-IR. In our study, a p < 0.05 was considered statistically significant. All statistical analyses were performed using SPSS version 19.0 (SPSS, Inc, Chicago, IL, USA).

## Results

Participants’ clinicodemographic characteristics are summarized in Table 1. In all, 24,088 people (13,638 men and 10,450 women) were considered for enrollment; of them, 5,061 who had MetS and met the inclusion criteria were included for analysis. Of the study participants, 3,253 were men (64.3%) and 1,808 were women (35.7%). The median age was 47 years for men and 56 years for women. The median HOMA-IR was 2.20 and 2.09, respectively, and prevalence of CKD was 11.31% and 15.93%, respectively, for men and women. Except WHtR, FBG, TC, LDL-C, all other variables were significantly different between the sexes.

**Table 1:** Basic characteristics of the study subjects

Variables	Total		Men		Women		p value
	(n = 5061)		(n = 3253)		(n = 1808)		
Age (year)	50	[43, 58]	47	[41, 54]	56	[50, 61]	< 0.001
Waist circumference (cm)	92	[87, 96]	94	[90, 98]	87	[82, 91]	< 0.001
Waist-to-height ratio	0.55	[0.53, 0.58]	0.55	[0.53, 0.58]	0.55	[0.53, 0.59]	0.13
BMI (kg/m^2^)	26.44	[24.72, 28.26]	26.82	[25.27, 28.53]	25.58	[23.84, 27.54]	< 0.001
SBP (mmHg)	133	[121, 144]	132	[121, 141]	135	[121, 148]	< 0.001
DBP (mmHg)	81	[73, 88]	84	[75, 90]	77	[70, 85]	< 0.001
MAP (mmHg)	99	[90, 106]	99.67	[91.67, 106.67]	96.67	[88.00, 104.92]	< 0.001
FBG (mmol/L)	5.67	[5.19, 6.32]	5.69	[5.18, 6.37]	5.65	[5.21, 6.23]	0.17
Total cholesterol (mmol/L)	5.32	[4.67, 6.01]	5.31	[4.67, 5.98]	5.34	[4.68, 6.05]	0.25
TG (mmol/L)	2.05	[1.52, 2.82]	2.23	[1.76, 3.10]	1.76	[1.21, 2.31]	< 0.001
LDL-C (mmol/L)	3.33	[2.75, 3.95]	3.31	[2.72, 3.95]	3.36	[2.79, 3.97]	0.22
HDL-C (mmol/L)	1.04	[0.93, 1.20]	0.99	[0.9, 1.12]	1.16	[1.04, 1.27]	< 0.001
Insulin (μIU/L)	8.3	[6.2, 11.2]	8.4	[6.2, 11.4]	8.0	[6.1, 10.8]	0.01
HOMA-IR	2.16	[1.57, 3.04]	2.20	[1.59, 3.11]	2.09	[1.53, 2.92]	0.001
eGFR (ml/min/1.73 m^2^)	98.21	[88.56, 106.17]	98.58	[88.04, 106.89]	97.75	[89.47, 104.41]	0.03
ACR (mg/g)	6.31	[3.73, 13.69]	5.38	[3.27, 11.41]	8.07	[5.01, 17.10]	< 0.001
Albuminuria, n (%)	612	(12.09%)	337	(10.36%)	275	(15.21%)	< 0.001
ACR, n (%)							< 0.001
normoalbuminuria (ACR < 30)	4449	(87.91%)	2916	(89.64%)	1533	(84.79%)	
microalbuminuria (ACR 30–299)	562	(11.10%)	311	(9.56%)	251	(13.88%)	
macroalbuminuria (ACR > = 300)	50	(0.99%)	26	(0.80%)	24	(1.33%)	
CKD, n (%)							< 0.001
Normal	4405	(87.04%)	2885	(88.69%)	1520	(84.07%)	
Stage 1	395	(7.80%)	202	(6.21%)	193	(10.67%)	
Stage 2	192	(3.79%)	117	(3.60%)	75	(4.15%)	
Stage 3	69	(1.36%)	49	(1.51%)	20	(1.11%)	
CKD stage 1 ~ 3, n (%)	656	(12.96%)	368	(11.31%)	288	(15.93%)	< 0.001

Continuous data are shown as medians [Q1, Q3] and compared using Mann–Whitney U test. Categorical data are shown as n (%) and compared using the Chi-square test

*BMI* body mass index, *SBP* systolic blood pressure, *DBP* diastolic blood pressure, *MAP* mean arterial pressur*e*, *FBG* fasting blood glucose, *TG* triglycerides, *LDL-C* low-density lipoprotein cholesterol, *HDL-C* high-density lipoprotein cholesterol, *HOMA-IR* homeostasis model assessment of insulin resistance, *eGFR* estimated glomerular filtration rate, *ACR* albumin/creatinine ratio, *CKD* chronic kidney disease

Table 2 revealed the correlation between the HOMA-IR level and cardio-metabolic risk factors. Except HDL-C, HOMA-IR was significant correlated with all the variables in men. In women, HOMA-IR was correlated with age, WC, WHtR, BMI, FBG, TG, HDL-C, insulin and ACR. Significant relationship between HOMA-IR and eGFR was only found in men.

**Table 2:** The correlation between HOMA-IR and cardio-metabolic risk factors in men and women

Variables	HOMA-IR index			
	Men		Women	
	Spearman’s coefficient	p value	Spearman’s coefficient	p value
Age (year)	−0.12	< 0.001	−0.06	0.01
Waist circumference (cm)	0.31	< 0.001	0.28	< 0.001
Waist-to-height ratio	0.28	< 0.001	0.23	< 0.001
BMI (kg/m^2^)	0.35	< 0.001	0.32	< 0.001
SBP (mmHg)	0.05	0.002	−0.04	0.10
DBP (mmHg)	0.04	0.01	−0.02	0.43
MAP (mmHg)	0.05	0.004	−0.04	0.12
FBG (mmol/L)	0.39	< 0.001	0.44	< 0.001
Total cholesterol (mmol/L)	0.10	< 0.001	0.01	0.60
TG (mmol/L)	0.10	< 0.001	0.10	< 0.001
LDL-C (mmol/L)	0.07	< 0.001	0.04	0.07
HDL-C (mmol/L)	0.03	0.09	−0.06	0.01
Insulin (mIU/L)	0.88	< 0.001	0.90	< 0.001
eGFR (ml/min/1.73 m^2^)	0.10	< 0.001	0.04	0.06
ACR (mg/g)	0.24	< 0.001	0.16	< 0.001

Spearman’s correlation was conducted to investigate the correlation between HOMA-IR and cardio-metabolic risk factors

*HOMA-IR* homeostasis model assessment of insulin resistance, *BMI* body mass index, *SBP* systolic blood pressure, *DBP* diastolic blood pressure, *MAP* mean arterial pressur*e*, *FBG* fasting blood glucose, *TG* triglycerides, *LDL-C* low-density lipoprotein cholesterol, *HDL-C* high-density lipoprotein cholesterol, *eGFR* estimated glomerular filtration rate, *ACR* albumin/creatinine ratio

The association of HOMA-IR and seven cardio-metabolic risk factors were shown in Table 3. HOMA-IR was significant associated with age, WHtR*10, eGFR, ACR in men. In women, HOMA-IR was only significant associated with WHtR*10.

**Table 3:** Association between cardio-metabolic risk factors and HOMA-IR

HOMA-IR			
Variables	Multivariable		
	B	95% CI	p value
Men			
Age (years)	−0.01	(−0.02, −0.001)	0.02
Waist-to-height ratio*10	1.06	(0.91, 1.22)	< 0.001
MAP	0.001	(−0.004, 0.01)	0.77
TG (mmol/L)	0.02	(−0.01–0.04)	0.14
HDL-C (mmol/L)	−0.10	(−0.42, 0.21)	0.52
eGFR (ml/min/1.73 m^2^)	0.01	(0.001, 0.01)	0.02
ACR (mg/g)	0.002	(0.001, 0.003)	< 0.001
Women			
Age (years)	0.02	(−0.02, 0.06)	0.42
Waist-to-height ratio*10	0.86	(0.24, 1.48)	0.01
MAP	−0.002	(−0.02, 0.02)	0.83
TG (mmol/L)	0.07	(−0.10, 0.23)	0.43
HDL-C (mmol/L)	−0.36	(−1.69, 0.97)	0.59
eGFR (ml/min/1.73 m^2^)	0.02	(−0.004, 0.05)	0.09
ACR (mg/g)	0.0004	(−0.002, 0.002)	0.69

*HOMA-IR* homeostasis model assessment of insulin resistance, *MAP* mean arterial pressur*e*, *TG* triglycerides, *HDL-C* high-density lipoprotein cholesterol, *eGFR* estimated glomerular filtration rate, *ACR* albumin/creatinine ratio

Table 4 presented the results of univariate and multivariable logistic analysis of the association between eight risk factors and prevalence of CKD. In men with MetS, the univariate logistic regression model indicated that CKD was significantly associated with all variables The multivariable logistic regression results revealed that except HDL-C, the other five risk factors were significantly associated with the prevalence of CKD. In women with MetS, age, WHtR*10, MAP had a significant association with CKD in both the univariate and multivariable logistic regression analyses.

**Table 4:** Association between cardio-metabolic risk factors and chronic kidney disease

Variables	Univariate			Multivariable		
	Odds ratio	95% CI	p value	Odds ratio	95% CI	p value
Men						
Age (years)	1.03	(1.02–1.04)	< 0.001	1.04	(1.03–1.05)	< 0.001
Waist circumstance (cm)	1.04	(1.02–1.06)	< 0.001	–	–	–
Waist-to-height ratio*10	2.37	(1.82–3.09)	< 0.001	1.57	(1.18–2.09)	0.002
MAP	1.06	(1.05–1.06)	< 0.001	1.06	(1.05–1.07)	< 0.001
TG (mmol/L)	1.07	(1.03–1.11)	< 0.001	1.10	(1.06–1.15)	< 0.001
HDL-C (mmol/L)	1.74	(1.07–2.83)	0.03	0.89	(0.52–1.53)	0.68
FBG (mmol/L)	1.26	(1.21–1.31)	< 0.001	–	–	–
HOMA-IR	1.22	(1.16–1.29)	< 0.001	1.21	(1.14–1.28)	< 0.001
Women						
Age (years)	1.04	(1.02–1.05)	< 0.001	1.03	(1.01–1.04)	0.001
Waist circumstance (cm)	1.05	(1.03–1.07)	< 0.001	–	–	–
Waist-to-height ratio*10	2.36	(1.83–3.05)	< 0.001	1.81	(1.38–2.38)	< 0.001
MAP	1.04	(1.03–1.05)	< 0.001	1.04	(1.03–1.05)	< 0.001
TG (mmol/L)	1.06	(0.99–1.13)	0.08	1.07	(1.00–1.16)	0.05
HDL-C (mmol/L)	1.35	(0.78–2.33)	0.28	0.81	(0.45–1.46)	0.48
FBG (mmol/L)	1.18	(1.12–1.25)	< 0.001	–	–	–
HOMA-IR	1.01	(0.99–1.03)	0.27	1.01	(0.99–1.02)	0.38

*MAP* mean arterial pressure, *TG* triglycerides, *HDL-C* high-density lipoprotein cholesterol, *FBG* fasting blood glucose, *HOMA-IR* homeostasis model assessment of insulin resistance

## Discussion

In this community-based and cross-sectional study, we investigated the association between insulin resistance and CKD in a Chinese population with metabolic syndrome, especially about the gender differences. Previous studies reporting that HOMA-IR was associated with CKD and microalbuminuria prevalence [7, 8, 21]. According to NHANES III, in US middle-aged individuals without diabetes, the odds ratio of CKD prevalence increased with HOMA-IR quartiles [7]. In a cohort study including Taiwanese adults aged  ≥ 65 years, those in the highest tertile of HOMA-IR had a higher prevalence of CKD compared with those in the lowest tertile (35.9% vs. 12.9%) [8]. Park et al. ‘s study demonstrated that Korean men with highest tertile of HOMA-IR had the highest risk of microalbuminuria [21]. In our multivariable linear regression analysis, HOMA-IR was significant associated with eGFR, ACR in men but not women. Our univariate and multivariable logistic regression analysis also revealed different results between men and women. In men, the prevalence of CKD was significantly associated with age, WHtR*10, MAP, TG and HOMA-IR value. In women, age, WHtR*10, MAP, but not HOMA-IR value were significantly associated with the prevalence of CKD. In the regression models of other studies, HOMA-IR value [8, 22, 23], high BP [8, 22], TG, FBG, low HDL-C level [8], and older age [22] were significantly associated with an increased odds ratio of CKD. In our study, HOMA-IR value had a significant association with eGFR, ACR and the prevalence of CKD only in men. Our result was similar to a Turkish study which showed that HOMA-IR was associated with a reduced eGFR in men, and the result was not present in women [24]. Zhao et al. performed a Mendelian randomization study and had the findings that fasting insulin was sex-specifically associated with CKD and unfavorable kidney function in men but not in women [25]. The sex-specific association of HOMA-IR and ACR was also demonstrated in studies on healthy Japanese adults and Caucasian adults with type 2 diabetes [^26^, ^27^]. We also observed that TG was significantly associated with CKD only in men. The result was similar to another Chinese study which Zhang et al. found that TG was associated with an increased risk of CKD only in male participants [28].

The sex disparity might be due to the involvement of sex hormones [29]. Estrogen has a favorable effect on insulin sensitivity; indeed, insulin sensitivity decreases after menopause and improves with hormone replacement therapy. The possible mechanisms include direct effects on insulin on glucose homeostasis, body composition, adipose tissue metabolism and distribution and proinflammatory markers. Other hormones related to IR include androgens, adiponectin, and dehydroepiandrosterone (DHEA) [29]. Sex hormones and their receptors also play a vital role in kidney injury and progression of renal disease. They have beneficial (mainly estrogens) or harmful (mainly androgens) effects through mechanisms such as oxidative stress, the renin–angiotensin system, inflammation, and fibrosis [3, 30]. Estrogen also prevents vascular injuries through direct and indirect action [31]. The aforementioned pathophysiological differences due to sex hormones may have cause the sex differences observed in our study and related studies.

Our study demonstrated that WHtR had higher OR than WC for CKD in both sexes. The result was similar with previous studies [32, 33]. WHtR is considered to be an indicator of central obesity. It corresponds to intraabdominal fat levels in computed tomography scan assessments [34]. Ashwell et al. strongly supported the use of WHtR as an index for obesity [35]. Many studies have demonstrated that WHtR had the highest association with cardiovascular disease risk factors [36]. Although obesity has been indicated to contribute to CKD and renal function decline in several studies [37–39], the pathophysiological mechanisms underlying this association have not been fully elucidated. Obesity has direct and indirect effects on the kidney disease. Obesity may indirectly promote CKD by increasing the risk of type 2 diabetes, hypertension, and atherosclerosis [40]. The direct pathogenesis includes renal hemodynamics change, increased inflammatory cytokines, altered adipokines, and increased growth factor [40–44]. Excess adiposity results in glomerular hyperfiltration, hypertrophy, and hypertension, leading to subsequent glomerulosclerosis and proteinuria. Excess adiposity also stimulates the production of proinflammatory and proatherogenic cytokines, such as Tumor Necrosis Factor -α and Interleukin-6, which are involved in CKD development. Adipose tissue secretes adipokines, including angiopoietins, vascular endothelial growth factor, cathepsins, leptin, adiponectin, and resistin. The alterations in the levels of adipokines, especially high leptin and low adiponectin levels, contribute to oxidative stress, increase sympathetic nervous system activity, cellular hypertrophy, extracellular matrix accumulation, proteinuria, and renal fibrosis.

### Strengths

First, our study analyzed a large, population-based sample, which gave high credibility to the results. The result also provided epidemiological data for CKD prevalence in the South China area. Second, we used the Chronic Kidney Disease Epidemiology Collaboration (CKD-EPI) equation to estimate GFR, which was more accurate than the Modification of Diet in Renal Disease Study (MDRD) equation [17].

### Limitations

Our study had some limitations. First, this is a cross-sectional design which is unable to investigate the causal relationship. Second, we did not consider menopausal status, so the influence of female hormones on renal function could not be investigated. We could not analyze the data in premenopausal and menopausal subgroups. Third, we used the HOMA-IR value instead of the hyperinsulinemic-euglycemic glucose clamp (HEGC) technique to estimate IR. Some studies have demonstrated that the HOMA-IR was not appropriate in participants with severely impaired or absent β-cell function [45].

## Conclusion

Our research adds to the growing evidence of gender differences in the associations between insulin resistance and CKD in population with metabolic syndrome. We found that HOMA-IR value was independently associated with CKD among men but not in women. These findings suggest that we should carefully evaluate the renal function progression for at-risk individuals with higher HOMA-IR value, particularly in men. Longitudinal studies in different populations and age groups are needed to prove our findings.

## Data Availability

The data analyzed in the present study are available from the corresponding author upon reasonable request.
